# SF3B4 downregulation restrains lung adenocarcinoma tumorigenesis via 5′ alternative splicing of KAT2A

**DOI:** 10.1038/s41598-023-50606-2

**Published:** 2024-01-02

**Authors:** Ailin Qu, Bo Han, Mengmeng Hua, Chune Wang, Tao Li

**Affiliations:** 1 Department of Clinical Laboratory, Qilu Hospital, Shandong University, Jinan, 250012 Shandong China; 2 Department of Pathology, Qilu Hospital, Cheeloo College of Medicine, Shandong University, Jinan, Shandong China; 3 Department of Oral and Maxillofacial Surgery, Qilu Hospital of Shandong University, Jinan, Shandong China; 4 Institute of Stomatology, Shandong University, Jinan, Shandong China; 5 Department of Respiratory Diseases, Qilu Hospital of Shandong University, No. 107, Culture West Road, Jinan, China

**Keywords:** Cancer, Cell biology, Molecular biology, Oncology

## Abstract

Aberrant expression of splicing factors, including SF3B4, plays a vital role in lung adenocarcinoma (LUAD). However, the impact of SF3B4 in the progression of LUAD has not been studied well. Here, we demonstrated the effects of SF3B4 in LUAD via apoptosis, proliferation, migration assays, etc. Gene manipulations confirmed the role of SF3B4 via KAT2A. SF3B4 was found to promote LUAD growth. Further studies found that, upon SF3B4 knockdown in LUAD cells, an alternative splice site occurred at the 5′-UTR of KAT2A, which led to the downregulation of KAT2A at both RNA and protein levels. Furthermore, the decrease in KAT2A expression partially reversed the effect of SF3B4 in promoting tumorigenesis. The axis SF3B4/ KAT2A was identified as a significant player in LUAD progression, shedding light on the therapeutic development in LUAD.

## Introduction

Lung adenocarcinoma (LUAD) is the most common subtype of non-small cell lung cancer (NSCLC), with a high incidence rate and low survival time^1^. Despite progress in surgical methods and therapeutic targets over the past years, no evident improvement has been achieved in the survival of LUAD patients^2^. This is primarily attributed to the complications associated with diverse mutations in LUAD. Hence, this unmet need creates a strong drive to uncover the mechanisms underlying the development of LUAD to improve patient outcomes.

Alternative splicing (AS) produces different mRNA splicing isomers from an mRNA precursor through different splicing methods. This produces isoforms of mRNA and protein variants from a single gene^3^, leading to different structural and functional properties in final protein products. Abnormal AS was shown to cause cancer^4,5^, and regulate cancer progression^6,7^. For example, SRSF1, a splicing factor, alternates the splicing of PTPMT1, regulating the resistance to radiotherapy in LUAD^8^.

RNA splicing is modulated by two spliceosomes, of which the major one functions through U2 snRNP, a particle consisting of the splicing factors SF3a, SF3b, and U2 snRNA^9^. SF3B4 is one of the SF3b complex components^10^. The SF3B4 haploinsufficiency or mutation was reported to be a major genetic factor that caused Nager syndrome^11^. Notably, dysregulation of SF3B4 expression has been implicated in multiple tumors. The expression of SF3B4 was shown to be increased in hepatocellular carcinoma compared to that in normal tissues^12,13^. In LUAD, Kim H reported that SF3B4 depletion slowed down the growth of LUAD cells via Ubiquitination factor E4B-mediated expression of p53/p21 and p27^14^. However, the research on the relationship between SF3B4 and LUAD is only at the beginning stage, especially on the effect and mechanism of SF3B4 alternative splicing on LUAD has not been studied well.

Here, we found that SF3B4 protein was upregulated in LUAD patients and played the role of oncogenes in the proliferation, migration, and invasion of LUAD. In addition, our results reveal that SF3B4 regulated LUAD progression via the alternative splicing of KAT2A for the first time.

## Results

### Aberrant expression of SF3B4 in LUAD

**Figure 1:**
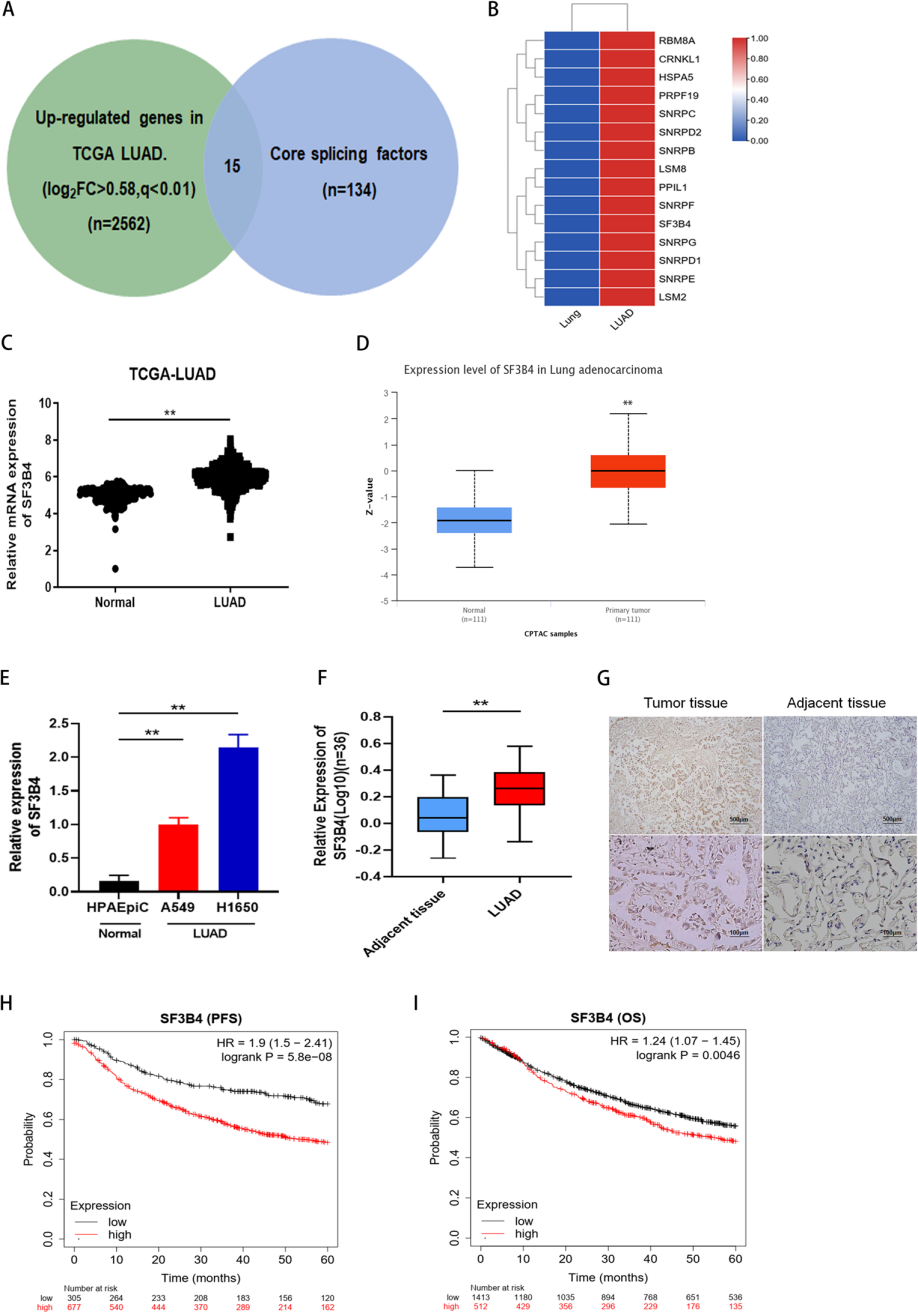
Aberrant expression of SF3B4 in LUAD. (**A**) Venn diagram showing the 15 genes correlated with upregulated genes in TCGA LUAD (n = 2562) and involved in cone splicing genes (n = 134). (**B**) Heatmap of selected genes in (**A**). (**C**) Relative mRNA expression of SF3B4 in LUAD and normal lung tissues from GTEX and TCGA and database. (**D**) Expression of SF3B4 protein in LUAD and norma lung tissues from CPTAC database. (**E**) SF3B4 mRNA levels in A549, H1650, and HPAEpiC cells, measured by qRT-PCR. (**F**) SF3B4 mRNA level in human lung tissues (n = 36), analyzed by qRT-PCR. (**G**) IHC staining of SF3B4 in human LUAD tissues and corresponding healthy tissues. (**H**,**I**) Kaplan–Meier analysis on the effects of SF3B4 on PFS and OS of LUAD patients. * *P* < 0.05, ** *P* < 0.01.

To search critical splicing factors involved in LUAD, overlapping analysis of 2562 upregulated genes and 134 core splicing factors revealed 15 potential candidates, including RBMBA, LSM8, and SF3B4 (Fig. 1A,B). To explore the effects of SF3B4 in LUAD, we examined the mRNA profile of SF3B4 in LUAD and normal lung tissues from the GTEX and TCGA databases (Fig. 1C). Then, we investigated the expression of SF3B4 protein in LUAD and normal lung tissues from the CPTAC database (Fig. 1D). Notably, the mRNA level of SF3B4 was higher in LUAD tissues and LUAD cells than in normal samples (Fig. 1E,F). These findings were further confirmed by comparing the results from the staining of SF3B4 in human tumor tissues to those in healthy tissues (Fig. 1G).

We performed a Kaplan–Meier analysis to explore whether SF3B4 contributed to LUAD progression. We observed that patients with high levels of SF3B4 had worse overall survival (OS) and poor progression-free survival (PFS) (Fig. 1H,I). Next, correlations of SF3B4 to clinicopathological features were analyzed. Importantly, SF3B4 expression was substantially correlated with the TNM stage (Table 1) in LUAD.

**Table 1:** Clinicopathological variables and SF3B4 expression in LUAD patients (n = 36).

Variable	Case No. (%)	SF3B4 expression		*P* -value
		Low (n = 12)	High (24)	
Gender				
Male	22 (61.1%)	6	16	0.472
Female	14 (38.9%)	6	8	
Age (years)				
≤ 60	17 (47.2%)	5	12	0.732
> 60	19 (52.8%)	7	12	
Smoking history				
Yes	20 (55.6%)	8	12	0.482
No	16 (44.4%)	4	12	
Differentiation				
Poor	27 (75.0%)	9	18	0.999
Moderate/well	9 (25.0%)	3	6	
TNM stage				
I/II	17 (47.2%)	9	8	0.032
III	19 (52.3%)	3	16	

### Promoting the role of SF3B4 in LUAD

**Figure 2:**
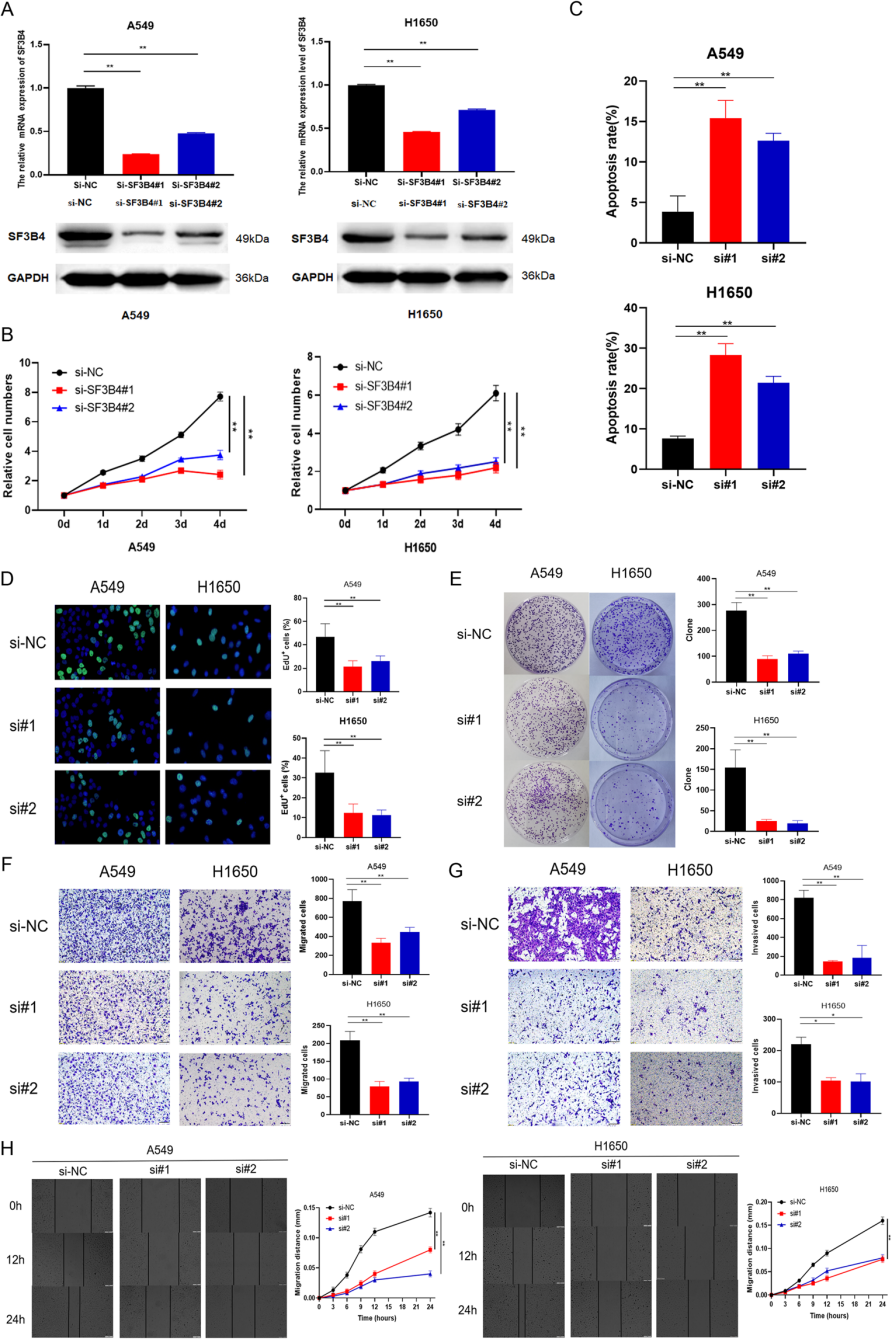
Effects of SF3B4 in proliferation, mobility and apoptosis of LUAD cells. (**A**) qRT-PCR and western blotting to confirm SF3B4 knockdown. (**B**) CCK8 assay on LUAD proliferation upon siRNAs transfection. (**C**) Apoptosis assay in LUAD cells. (**D**) EdU assay on proliferation capacity of LUAD cells upon siRNAs transfection. (**E**) Colony formation of LUAD cells upon siRNAs transfection. (**F**,** G**) Migration and invasion assays on LUAD cells upon siRNAs transfection. (**H**) Wound-healing assay on LUAD cells upon siRNAs transfection. * *P* < 0.05, ** *P* < 0.01. Original blots/gels are presented in Supplementary Raw data.

To explore the role of SF3B4 in LUAD, we transfected siRNA targeting SF3B4 into wild-type LUAD cells. The efficiency of SF3B4 knockdown was validated in transcription and translation levels (Fig. 2A). SF3B4 knockdown significantly suppressed the proliferation of LUAD cells (Fig. 2B,D). Besides, SF3B4 knockdown significantly inhibited the formation of colonies derived from A549 and H1650 cells, respectively (Fig. 2E). Furthermore, we found that the knockdown of SF3B4 decreased the capacity to migrate and invade LUAD cells (Fig. 2F,G). Consistently, the knockdown of SF3B4 inhibited the capacity of LUAD cells in wound healing (Fig. 2H). Futhermore, we showed that SF3B4 knockdown induced apoptosis in LUAD cells in vitro (Fig. 2C, Supplementary Fig. 1). These data suggest that targeting SF3B4 could be an effective treatment for LUAD.

### SF3B4 promotes LUAD tumor growth

**Figure 3:**
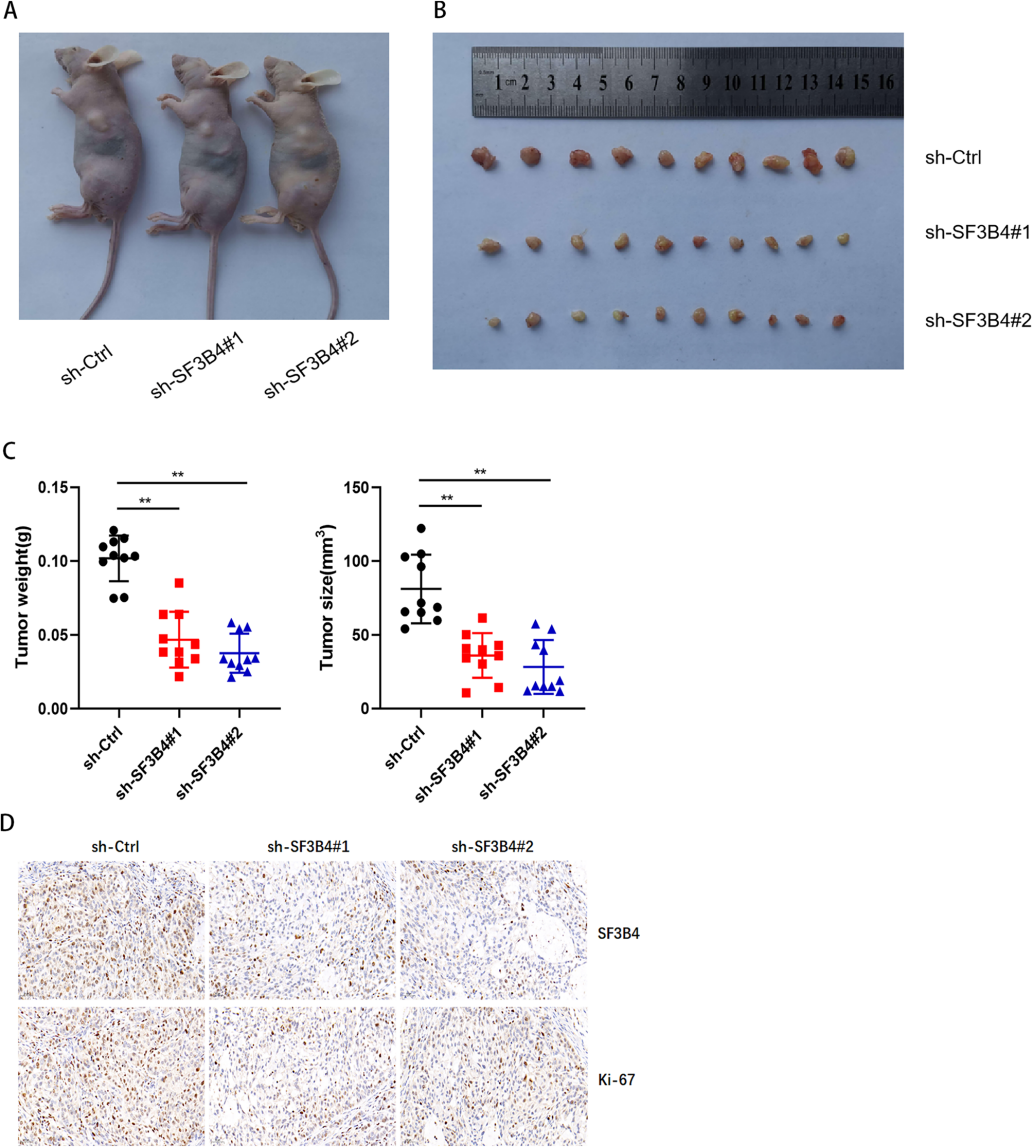
Effect of SF3B4 on LUAD growth in vivo. (**A**) Images of mice with tumors (n = 5). (**B**) Tumors derived from sh-SF3B4 cells or control cells. (**C**) Tumor weights and size. (**D**) IHC staining results for SF3B4 and Ki-67 in tumor tissues from a mouse model. * *P* < 0.05, ** *P* < 0.01.

To examine the effects of SF3B4 in vivo, we constructed the cell lines with a knockdown of SF3B4 and a cell line with a control vector. We subcutaneously inoculated sh-SF3B4 and sh-Ctrl cells in mice. After 3 weeks, we found dramatic decreases in tumor size and weights from the mice-bearing tumors derived from SF3B4 knockdown cells compared to the control (Fig. 3A–C). Besides, we quantified the levels of SF3B4 and Ki-67 with immunohistochemical staining in this nude mouse xenograft model. The level of SF3B4 was reduced in the SF3B4 knockdown group (Fig. 3D). The intensity of Ki-67 was lower in tumors derived from SF3B4 knockdown cells than that derived from control (Fig. 3D). In summary, the results demonstrated a promoting role of SF3B4 on LUAD.

### KAT2A is an essential downstream effector of SF3B4

**Figure 4:**
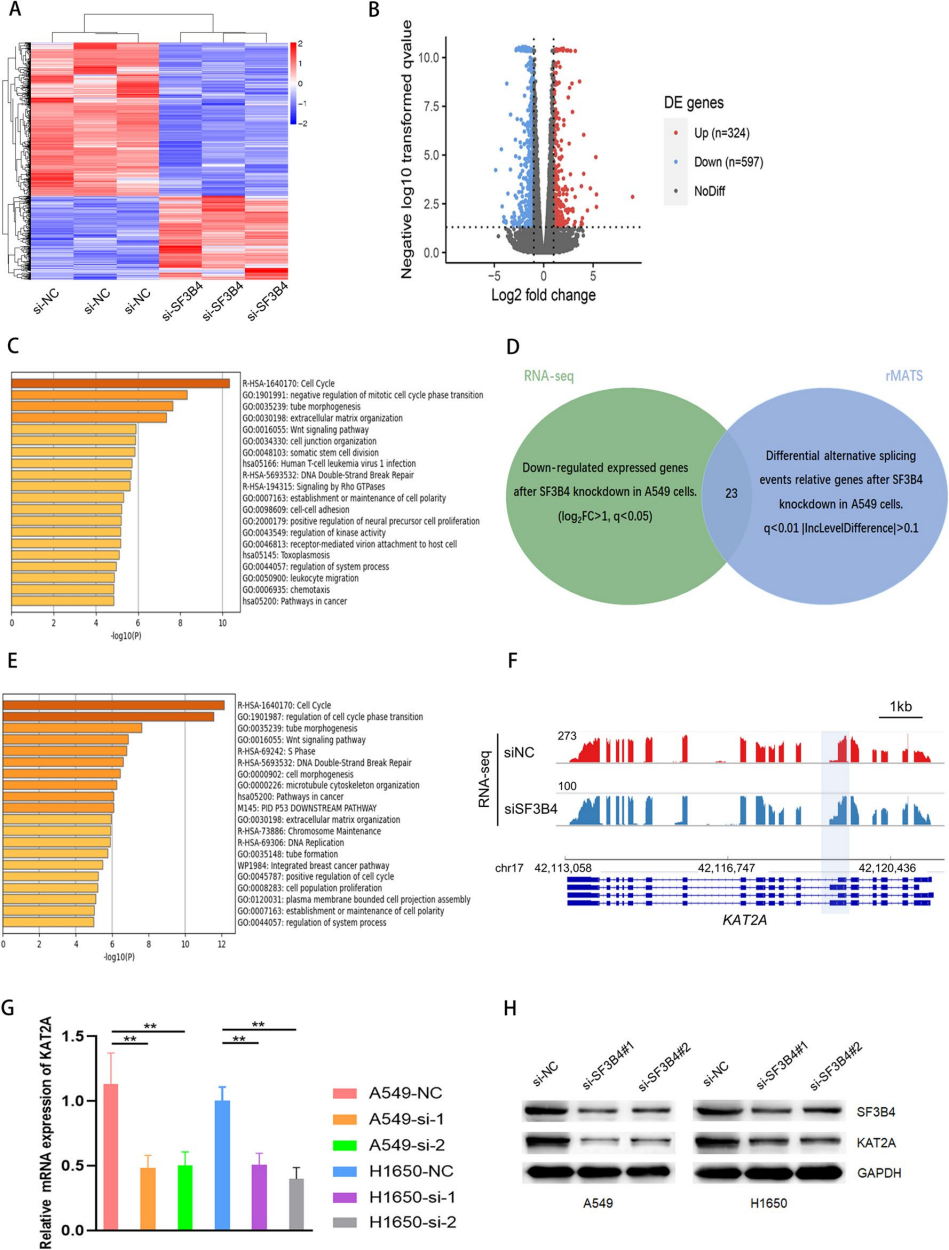
KAT2A is a downstream target of SF3B4. (**A**,**B**) Heatmap and volcano plots of DEGs from RNA-seq in A549 cells with or without SF3B4 knockdown. (**C**) GO analysis of DEGs. (**D**) Venn diagram of 3022 genes related to differential AS events upon knockdown of SF3B4. (**E**) GO analysis of downregulated DEGs (**F**) RNA-seq reads mapping to KAT2A in A549 cells upon knockdown of SF3B4. (**G**,**H**) qRT-PCR and western blotting of KAT2A levels upon SF3B4 knockdown in A549 cells. * *P* < 0.05, ** *P* < 0.01. Original blots/gels are presented in Supplementary Raw data.

RNA-seq was performed in A549 cells with or without SF3B4 knockdown to explore the pathway through which SF3B4 drove the growth and development of LUAD. The profile of differentially expressed genes (DEGs) was plotted (Fig. 4A,B). We found 324 upregulated and 597 downregulated genes. Gene analysis was performed to depict the biological functions of these DEGs. Interestingly, the enriched biological pathways are involved in regulating the cell cycle and the negative regulation of phase transitions in the mitotic cell cycle (Fig. 4C). Exploring the genes that are down-regulated after SF3B4 knockdown could provide valuable insights. For downregulated genes upon SF3B4 knockdown, the enrichment of biological pathways was DNA double-strand break repair, cell cycle regulation, and DNA replication in downregulated genes (Fig. 4E). Interestingly, 23 genes were identified to have both downregulated expressed genes and were involved in the differential AS events in A549 cells (Fig. 4D). We then performed visual analysis on the identified genes, and rMATs found that the 5′ end of KAT2A had variable splicing. An A5SS variable splicing event occurred in the annotated region of the shadow. When SF3B4 expression is interfered with si-SF3B4, a long exon is generated at the 5′ variable splicing site of KAT2A. This results in the transcription containing the long exon no longer encoding. This reduces the expression of KAT2A protein. (Fig. 4F). To further explore the role of SF3B4 in regulating KAT2A, the changes in the KAT2A level were confirmed by qRT-PCR and Western blotting upon SF3B4 knockdown in LUAD cells, including A549 and H1650. Both mRNA and protein levels of KAT2A were significantly downregulated upon SF3B4 knockdown (Fig. 4G,H). These results suggest that KAT2A is a target of SF3B4 in LUAD.

### SF3B4 leads to AS of KAT2A

**Figure 5:**
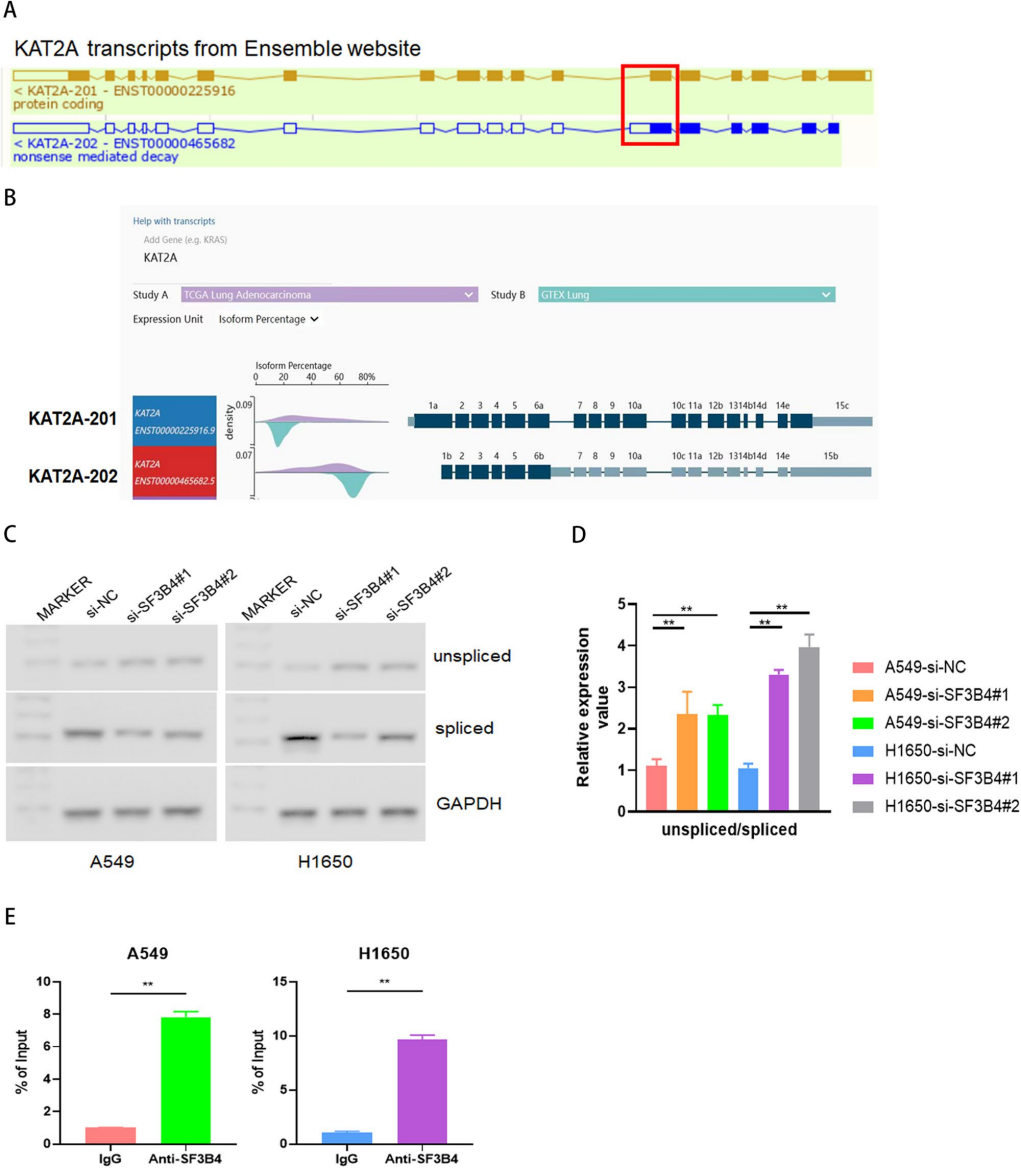
SF3B4 facilitates efficient splicing of KAT2A. (**A**) Schematic diagram on two splicing variants of KAT2A mRNA transcript. (**B**) Relative mRNA levels of KAT2A splicing variants in LUAD tissues (purple) and normal controls (green) from the TCGA and GTEX databases. (**C**,**D**) Relative mRNA levels of KAT2A transcripts in LUAD cells upon knockdown of SF3B4. (**E**) RIP-PCR in A549 and H1650 cells. * *P* < 0.05, ** *P* < 0.01.

We further analyzed the splice variants of KAT2A mRNA with an ensemble genome browser to explore if SF3B4 regulated the levels of KAT2A through AS. We found that a long exon was produced in the unspliced transcript of KAT2A-202, different from the spliced transcript of KAT2A-201 (Fig. 5A). KAT2A-201 mRNA level was increased than that of KAT2A-202 in LUAD cells. Furthermore, we analyzed the levels of KAT2A-201 and KAT2A-202 transcripts from both LUAD tissues and normal controls in TCGA and GTEX databases. We found that the expression of KAT2A-201 transcripts in LUAD was relatively higher than that of KAT2A-202, while KAT2A-202 was relatively higher in normal lung tissue (Fig. 5B). RT-PCR results verified the changes in the levels of KAT2A-201 and KAT2A-202. We observed that upon SF3B4 knockdown, the level of the unspliced transcript variant was significantly increased, and that of the spliced transcript variant was significantly reduced (Fig. 5C,D). Interestingly, RIP-PCR data showed that the mRNA level of KAT2A in SF3B4 precipitates was significantly increased than that in the IgG group (Fig. 5E). This suggests that SF3B4 regulates the KAT2A via A5SS.

### Targeting KAT2A in LUAD

**Figure 6:**
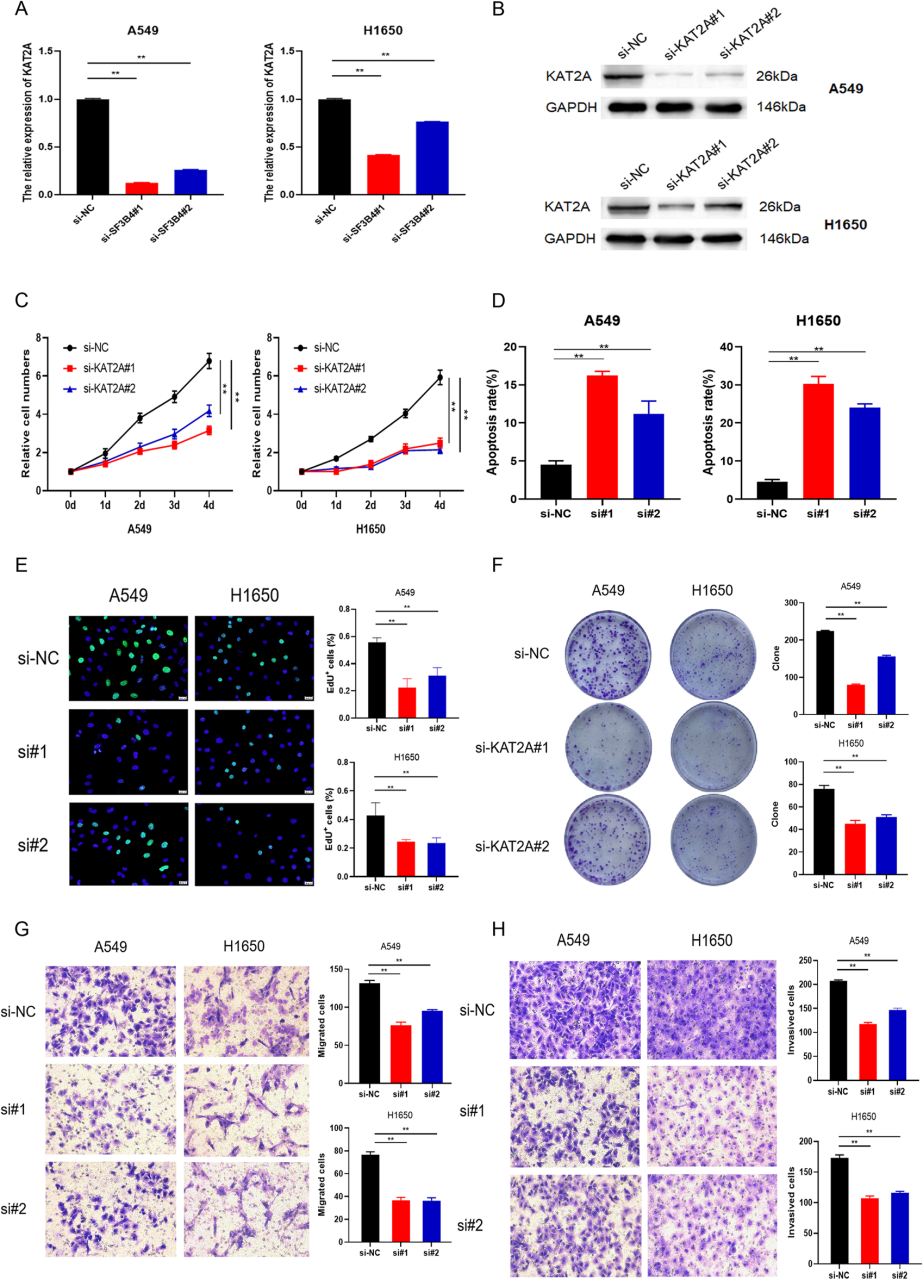
Effects of KAT2A on proliferation and mobility of LUAD. (**A**,**B**) qRT-PCR and western blotting on the efficiency of KAT2A knockdown by targeted siRNAs in LUAD. (**C**) CCK-8 test on cell proliferation. (**D**) Apoptosis assay on A549 and H1650 cells with or without KAT2A knockdown. (**E**) EdU test on cell proliferation. (**F**) Clonogenic assay on A549 and H1650 cells. (**G**,** H**) Transwell assays on A549 and H1650 cells. * *P* < 0.05, ** *P* < 0.01. Original blots/gels are presented in Supplementary Raw data.

To evaluate the role of KAT2A alone in LUAD cells, we transiently silenced KAT2A with siRNA transfection into LUAD cells. The knockdown of KAT2A was confirmed (Fig. 6A,B). The analyses revealed that KAT2A knockdown decreased cell viability and colony formation and increased cell apoptosis (Fig. 6C–F, Supplementary Fig. 2). Transwell analysis results showed that knockdown of KAT2A reduced the invasion and migration capacities of LUAD cells (Fig. 6G–H).

**Figure 7:**
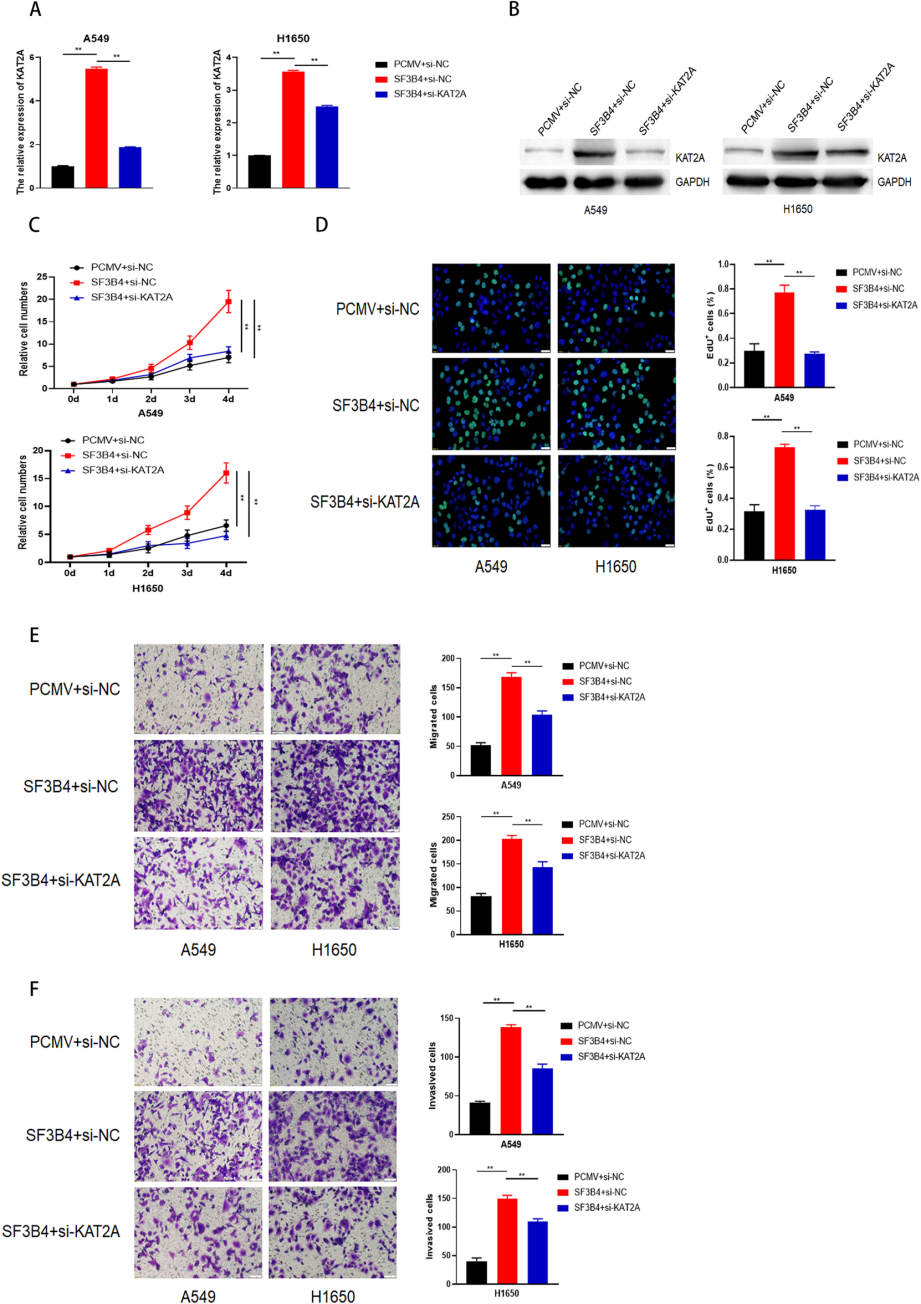
Effects of KAT2A on proliferation and mobility of LUAD cells with SF3B4 overexpression. (**A**,**B**) qRT-PCR and western blotting on the efficiency of KAT2A knockdown in LUAD cells with or without SF3B4 overexpression. (**C**) CCK-8 test on cell proliferation. (**D**) EdU test on cell proliferation of A549 and H1650 cells. (**E**,**F**) Transwell assays on A549 and H1650 cells upon KAT2A knockdown, with or without overexpression of SF3B4. * *P* < 0.05, ** *P* < 0.01. Original blots/gels are presented in Supplementary Raw data.

Furthermore, rescue experiments were performed to confirm the effect of KAT2A in SF3B4-regulated malignancy of LUAD. We transfected si-NC or KAT2A-targeted siRNA into LUAD cells with SF3B4 overexpression (Fig. 7A,B). We observed that proliferation rate, migration, and invasion capacities in LUAD cells enhanced with SF3B4 overexpression were impaired upon the KAT2A knockdown (Fig. 7C–F). These findings suggest the effects of KAT2A in the SF3B4-modified oncogenesis of LUAD.

## Discussion

Emerging evidence has manifested that splicing factor SF3B4 binds to U2 snRNP, which regulates the AS of premature mRNA^15^. SF3B4 is an oncogenic driver in multiple tumor types. However, it is a tumor-suppressor in pancreatic cancer^16–19^. We showed increased levels of SF3B4 in LUAD tissues and cells. The upregulated level of SF3B4 was associated with a poor prognosis of LUAD. Furthermore, we observed that SF3B4 knockdown suppressed proliferation and mobility and induced apoptosis in LUAD cells. Meanwhile, we proved that the SF3B4 knockdown slowed tumor growth in a mouse model. Our investigation showed that SF3B4 exhibited oncogenic properties in LUAD, in line with other studies^14^.

Understanding the underlying mechanisms of SF3B4 as an oncogene requires further studies. In cervical cancer, Li et al. proposed that the knockdown of SF3B4 decreased the splicing efficiency of SPAG5 via retained intron, leading to a reduction in SPAG5 expression^20^. In hepatocellular carcinoma, the SF3B4 knockdown induced the inactivation of p27 by skipped exon, alternating the splicing of KLF4^9^. Since the splicing effect of SF3B4 has not been studied well in LUAD, RNA-seq was performed to screen AS events. Through rMAT analysis and RT-PCR validation, we found that an alternative splice event occurred at the 5 'end of the KAT2A gene, producing a long exon. The transcript containing the long exon does not encode, resulting in the downregulation of KAT2A after SF3B4 knockdown in LUAD. Therefore, for the first time, we found that SF3B4 regulated LUAD progression via the alternative splicing of KAT2A.

The 5′ untranslated region (5′UTR) of a mature mRNA is involved in gene regulation at the post-transcriptional level. Within a given transcriptional unit, AS is one event that induces changes to the 5′UTR sequence^21^, leading to differences in protein variant output. Selecting 5′ splice-sites (5′SS) in AS plays essential roles in gene regulation, including cell proliferation, invasion, and apoptosis^22–24^. KAT2A, a member of the N-acetyltransferase superfamily, was identified as a histone acetyltransferase binding to acetyl-CoA^25,26^. Various studies have indicated that KAT2A is an epigenetic oncogene in several malignancies^27–29^. Besides, KAT2A has been reported to promote stem-like cell propagation in leukemia and regulate the resistance to tamoxifen in breast cancer^30^. However, little was known about the effects of KAT2A in LUAD. Here, we confirmed that KAT2A knockdown decreased the capacities of LUAD cells to proliferate, invade, and migrate. The KAT2A knockdown also impaired the phenotypic changes induced upon overexpression of SF3B4.

Taken together, we explored the effects of SF3B4 in LUAD and possible mechanisms. To the best of our knowledge, SF3B4 enhanced LUAD proliferation, invasion, and migration through the alternative 5′ splice Site (A5SS) of KAT2A for the first time. The KAT2A knockdown impaired the function of SF3B4 in LUAD cells. The SF3B4/ KAT2A axis could be a potential pathway to treat LUAD.

## Materials and methods

### Bioinformatics

The data for upregulated genes in LUAD was from GEPIA (http://gepia.cancer-pku.cn/). Heatmap data of cone splicing factors was from UCSC Xena (https://xena.ucsc.edu/). Expression of SF3B4 in normal tissues and LUAD was from TCGA and GTEX. Kaplan–Meier analysis was used to analyze the correlation of SF3B4 and OS.

### Cell lines and culture

HPAEpiC, A549, and H1650 were purchased from CBTCCCAS (Shanghai, China). All cells were cultured in RPMI 1640 medium (Gibco, USA) with 10% fetal bovine serum (FBS, GeneDEPOT Barker, TX, USA), 100 U/mL penicillin, and 100 µg/mL streptomycin (Biowest, Nuaillé, France) at 5% CO_2,_ 37 °C and 95% humidity.

### LUAD tissue

Primary LUAD tissues and adjacent normal lung tissues were harvested from patients in Qilu Hospital of Shandong University (Jinan, China) from May 2022 to October 2022. Prior to surgery, no patients received radiotherapy or chemotherapy. Tissues were immediately immersed in liquid nitrogen and then stayed at − 80 °C. Consent was obtained from subjects. The studies were approved by the Ethics Committee of Qilu Hospital of Shandong University (KYLL-202205-043-1). The clinic pathological features are summarized in Table 1.

### RNA isolation and qPCR

Trizol (Invitrogen) reagent was used for total RNA extraction. A master mix kit, PrimeScript RT (Takara, RR037A), was used for cDNA synthesis. SYBR Green (Takara, RR420A) was used for qRT-PCR. For sequences for primers, see Supplementary Table 1.

### Western blotting

Cell lysis was done in RIPA buffer (Beyotime Bio, Cat# P0013). The protein amount was measured with a Pierce BCA kit (Merck Millipore, Cat# 71,287). Protein samples were separated via SDS-PAGE and transferred to the PVDF membrane (Immobilon-P; Millipore, Billerica, MA, USA). After blocking with 5% BSA or fat-free milk (Amresco), the PVDF membrane was probed with primary antibodies at 4 °C overnight and then washed in TBST three times (15 min/wash). The membrane was then incubated with secondary antibodies (1:4000) at room temperature for 1 h. The antibodies information are SF3B4 (1:8000, Proteintech, 10,482-1-AP), KAT2A (1:1000, Proteintech, 66,575-1-Ig), and GAPDH (1:8000, Sigma–Aldrich, ABS16). Enhanced chemiluminescence reagent (Amersham, RPN2232) was used to expose protein bands on ImageQuant LAS 4000 (GE Healthcare Life Sciences).

### RNA interference and lentiviral infection

Open reading frames of KAT2A and SF3B4 were from Vigenebio. Small interference RNAs were from GenePharma (Shanghai, China). shRNAs were constructed from vector pLKO.1-TRC. Lentivirus particles were packaged with pMD2.G and psPAX2 in HEK293 cells. LUAD cells were infected with lentivirus for 24 h. LUAD cells were then screened for 7 days in the presence of puromycin (2 μg/ml). See the sequences in Supplementary Table 2.

### RNA sequencing

A549 cells were used for RNA sequencing (RNA-seq). Total mRNA was isolated with TRIzol reagent. Three replicates were produced for RNA sequencing for each sample based on the Shanghai Biotechnology Co platform. For raw sequencing data, see GSE222598 in the GEO database. An adjusted *p*-value (*p* < 0.05) and FDR < 0.01 were used as cut-offs for significantly differential expression.

### Proliferation measurements

To examine cell proliferation, 3000 cells were seeded into 96-well plates. Cell viability was quantified with a CCK-8 assay kit (Invitrogen, Carlsbad, California, USA). Absorbance at 450 nm was read with a spectrophotometer (Thermo Scientific NanoDrop-2000).

Edu (Beyotime, C0071s) staining was done based on the manufacturer’s protocol. Cells (1.5 × 10^4^) in 96-well plates were incubated with Edu (1:1000 dilution) for 2 h. Cells fixation was done with 4% of paraformaldehyde. EdU immunofluorescence staining was performed using the EdU Kit (Thermo Fisher, USA) according to the manufacturer's protocol T^31^.

### Colony formation

On day 1, cells (1 × 10^3^/well) were seeded into a 6-well plate, followed by medium change every 2 days. On day 10, colony fixation was done with methanol. Then, colonies were stained with 0.6% crystal violet and counted in Photoshop (CC 2022).

### Invasion and migration

These assays were performed in transwell plates with transwell filters with 8.0 μm pore size (Corning, Cambridge, MA, USA). siRNA-transfected LUAD cells were harvested 24 h after transfection. Then, cells were washed and seeded into 24-well chambers with or without a coat of matrigel for invasion, but migration was unnecessary. Cells (1 × 10^5^ per well) were seeded into the upper chamber in a serum-free medium. The lower chamber was filled with medium with 10% FBS. Cells were allowed to migrate or invade through pores for 12–24 h at 37 °C. At the endpoint, cells in the lower chamber were fixed in 4% paraformaldehyde. Then, cell staining was performed with 0.1% crystal violet (Sigma, C3886) at room temperature for 5 min. Cell counting was conducted under a microscope (IX71, Olympus).

### Wound healing

Cells (1 × 10^5^/well) were seeded as monolayers in a 24-well plate. When cells were 100% confluent, a scratch line was created with a 20 µl pipette tip. Then, a medium without serum was used for cell culture. The distance between the two edges of the scratch line was monitored at 0, 12, and 24 h under a microscope (IX71, Olympus).

### Immunohistochemistry

The tissue specimen was fixed with formalin and embedded with paraffin. Then, the tissue was sliced into approximately 5 μm thickness. The tissue slice was deparaffinized with xylene, rehydrated with graded ethanol, and then incubated with 3% H_2_O_2_ to inhibit endogenous peroxides activity. The tissue slice was first incubated with a primary antibody at 4 °C overnight, followed by incubation with a secondary antibody for 1 h in the dark. The antibodies were anti-SF3B4 (1:200, Proteintech, 10,482–1-AP) and anti-Ki-67 (1:600, Cell Signaling Technology, 9449 T). In the end, 3,3′-diaminobenzidine tetrachloride (Sigma) chromogen solution was used to detect the amount of bound antibody. Samples were counterstained with hematoxylin, dehydrated, and mounted.

### Apoptosis and cell cycle

LUAD cells were seeded as monolayers in a 6-well plate. After transfection with shSF3B4 or shControl, cells were cultured for 72 h. Cells were then harvested and stained with FITC‐Annexin V and Propidium Iodide (BestBio, 4101-1). Flow cytometry (FACScan®; BD Biosciences) was used for analysis^32^.

### Animal study

The study was performed based on protocol (No. SDULCLL2022-06-05) approved by the Animal Care and Use Committee of Shandong University. All methods followed relevant guidelines and regulations and were reported based on ARRIVE guidelines (https://arriveguidelines.org). BALB/c male nude mice (4 weeks old) were obtained from the Shanghai Laboratory Animal Center of the Chinese Academy of Sciences (Shanghai, China). Animals were randomized into two groups. One group was inoculated subcutaneously with control cells; the other was inoculated with shSF3B4 cells (5 million cells per mouse). Tumor size was calculated based on: Tumor volume = L × W^2^ × π/6, in which W represents the shortest diameter, and L represents the longest diameter. At the endpoint, mice were euthanized with CO_2_.

### RNA immunoprecipitation

RIP Kit (P0101, Geneseed Biotech, Guangzhou) was used to capture the antigen after the magnetic beads were connected to the KAT2A antibody or IgG antibody. qRT-PCR was performed to detect the KAT2A mRNA level.

### Statistical analysis

Statistics were performed with SPSS (version 20.0, Chicago, IL). Data plotting was done with GraphPad 7.0 (La Jolla, CA, USA). The comparison was conducted by unpaired two-tailed t-tests. All experiments had three independent replicates. Data were represented as mean ± SD. *p* < 0.05 is statistically significant. The χ^2^-test was used to assess the relationship between expression of SF3B4 and clinical factors.

## Ethics statement

All methods were carried out per the ethical standards of the responsible committee on human experimentation or with the Helsinki Declaration of 1975. The studies were approved by the Ethics Committee of Qilu Hospital of Shandong University (KYLL-202205-043-1). Informed consent was obtained from patients.

## Supplementary Information

Supplementary Information.

## Data Availability

The datasets generated and analyzed during the current study are available from the corresponding author upon reasonable request.

## Abbreviations

AS: Alternative splicing
CBTCCCAS: Cell Bank of Type Culture Collection of Chinese Academy of Sciences
CPTAC: Clinical Proteomic Tumor Analysis Consortium
DEGs: Differentially expressed genes
FBS: Fetal bovine serum
GAPDH: Glyceraldehyde 3-phosphate dehydrogenase
GEO: Gene Expression Omnibus
GEPIA: Gene Expression Profiling Interactive Analysis
GTEX: Genotype-Tissue Expression
KAT2A: Lysine acetyltransferase 2A
KLF4: Krüppel-like factor 4
LUAD: Lung adenocarcinoma
mRNA: Messenger ribonucleic acid
OS: Overall survival
PFS: Progression-free survival
PTPMT1: Protein tyrosine phosphatase mitochondrial 1
PVDF: Polyvinylidene fluoride
RBMBA: Rugosity and biofilm structure modulator A
RT-PCR: Reverse transcription-polymerase chain reaction
SF3B4: Splicing factor 3B subunit 4
siRNA: Short interfering RNA
snRNA: Small nuclear RNA
snRNP: Small nuclear ribonucleoproteins
SPAG5: Sperm-associated antigen 5
SRSF1: Serine/arginine-rich splicing factor 1
TCGA: The Cancer Genome Atlas
UTR: Untranslated region
