# Correction: SF3B4 downregulation restrains lung adenocarcinoma tumorigenesis via 5′ alternative splicing of KAT2A

**DOI:** 10.1038/s41598-025-33009-3

**Published:** 2026-01-05

**Authors:** Ailin Qu, Bo Han, Mengmeng Hua, Chune Wang, Tao Li

**Affiliations:** 1 Department of Clinical Laboratory, Qilu Hospital, Shandong University, Jinan, 250012 Shandong China; 2 Department of Pathology, Qilu Hospital, Cheeloo College of Medicine, Shandong University, Jinan, Shandong China; 3 Department of Oral and Maxillofacial Surgery, Qilu Hospital of Shandong University, Jinan, Shandong China; 4 Institute of Stomatology, Shandong University, Jinan, Shandong China; 5 Department of Respiratory Diseases, Qilu Hospital of Shandong University, No. 107, Culture West Road, Jinan, China

Correction to: *Scientific Reports* 10.1038/s41598-023-50606-2, published online 02 January 2024

The original version of this Article contained an error in Figure 3D where portions of the images for the experimental groups labeled “SF3B4 + sh-SF3B4#1” and “SF3B4 + sh-SF3B4#2” were mistakenly repeated due to incorrect labeling of the image files during data assembly. The original Figure 3 and accompanying legend appear below.

**Fig. 3:**
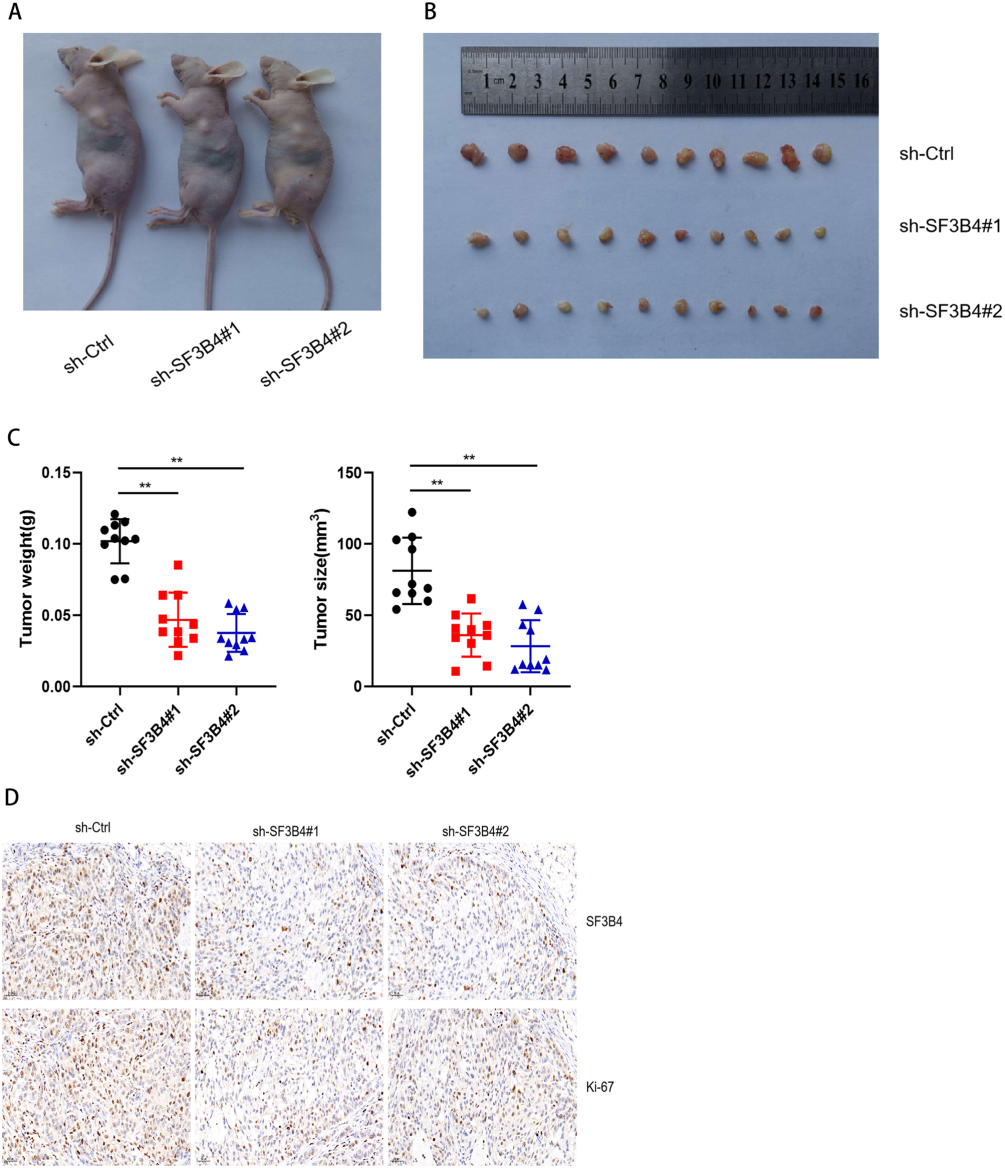
Effect of SF3B4 on LUAD growth in vivo. (**A**) Images of mice with tumors (n = 5). (**B**) Tumors derived from sh-SF3B4 cells or control cells. (**C**) Tumor weights and size. (**D**) IHC staining results for SF3B4 and Ki-67 in tumor tissues from a mouse model. * *P* < 0.05, ** *P* < 0.01.

The original Article has been corrected.

