# Supplementary material for: SF3B4 downregulation restrains lung adenocarcinoma tumorigenesis via 5′ alternative splicing of KAT2A
Source: Sci Rep. 2024 Jan 2;14:30. doi: 10.1038/s41598-023-50606-2 (PMC10762244; doi:10.1038/s41598-023-50606-2)

**SF3B4 downregulation restrains lung adenocarcinoma tumorigenesis via 5 'alternative splicing of KAT2A**

**Supplementary Figure1**

Apoptosis assay on A549 and H1650 cells with or without SF3B4 knockdown.


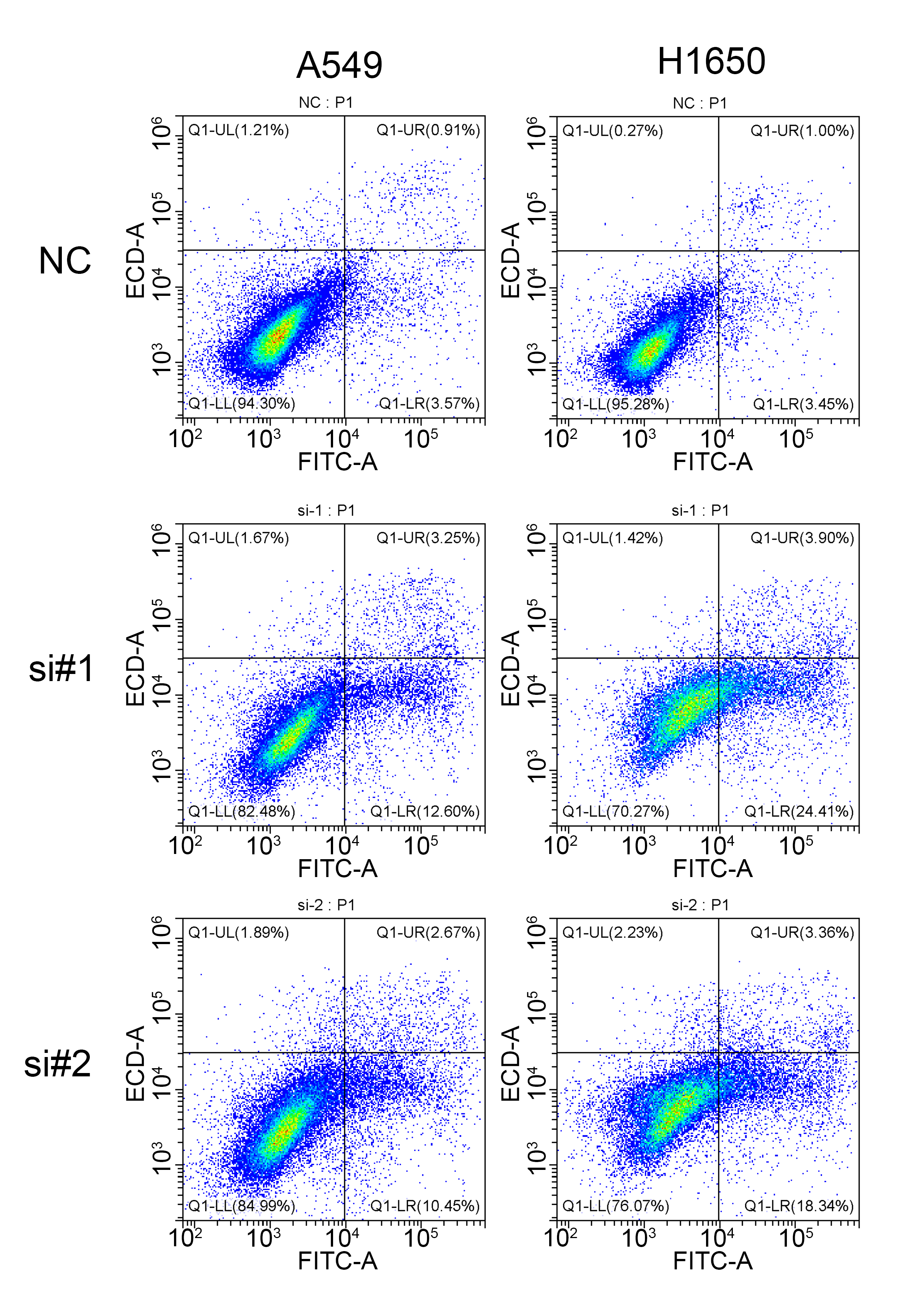


**Supplementary Figure2**

Apoptosis assay on A549 and H1650 cells with or without KAT2A knockdown


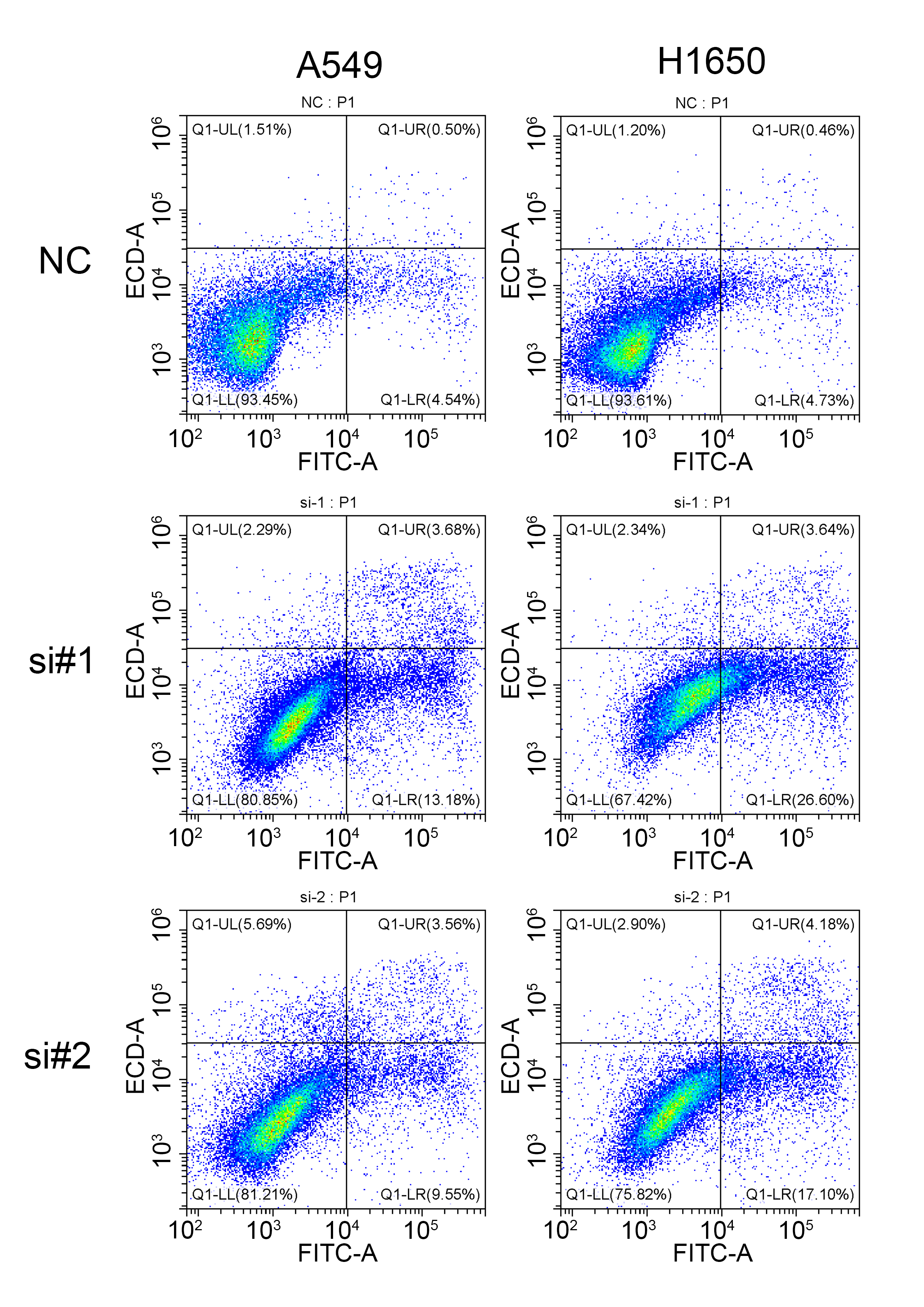


**Supplementary Table 1. Primers sequences**

| Gene | Sequence (5’-3’) |
| --- | --- |
| SF3B4-F | AGTCAACACCCACATGCCAA |
| SF3B4-R | CACCCGTATTGGCTTCCCAT |
| GAPDH-F | GGTCTCCTCTGACTTCAACA |
| GAPDH-R  KAT2A-201-F  KAT2A-201-R  KAT2A-202-F  KAT2A-202-R | GTGAGGGTCTCTCTCTTCCT  CCAACTCCGACTCCTGCAC  ACTTGCGCCTTCCTCTGACT  GTAAGGCTCCTTCTGGCCTA  ATGGGCAAGTCTGCCAGGTA |

**Supplementary Table 2**. siRNAs sequences

| Gene | | Sequence (5’-3’) | |
| --- | --- | --- | --- |
| si-SF3B4#1 | GGAUGAGAAGGUUAGUGAATT | |  |
| si-SF3B4#2 | GCACCAAGGCUAUGGCUUUTT | |  |
| si-KAT2A#1  si-KAT2A#2 | CAAGATCGAAGATGAAGAA  TCAAAGACCTCATCAATGA | |  |
| si-RNA-control | UUCUCCGAACGUGUCACGUTT | |  |
| sh-SF3B4-F1 | CCGGCCCTGAGATTGATGAGAAGTTCTCGAGAACTTCTCATCAATCTCAGGGTTTTTG | |  |
| sh-SF3B4-R1 | AATTCAAAAACCCTGAGATTGATGAGAAGTTCTCGAGAACTTCTCATCAATCTCAGGG | |  |
| sh-SF3B4-F2 | CCGGGGATGAGAAGGTTAGTGAACCCTCGAGGGTTCACTAACCTTCTCATCCTTTTTG | |  |
| sh-SF3B4-R2  sh-SF3B4-F(ov)  sh-SF3B4-R(ov) | AATTCAAAAAGGATGAGAAGGTTAGTGAACCCTCGAGGGTTCACTAACCTTCTCATCC  GCGAATTCGAAGTATACCTCGAGGCCACCATGGCTGCCGGGCCGATCTA  ATCGCAGATCCTTGGATCCTTACTGAGGGAGAGGGCCTCGAAGTGG | |  |

**WB original images**

**Figure 2A**


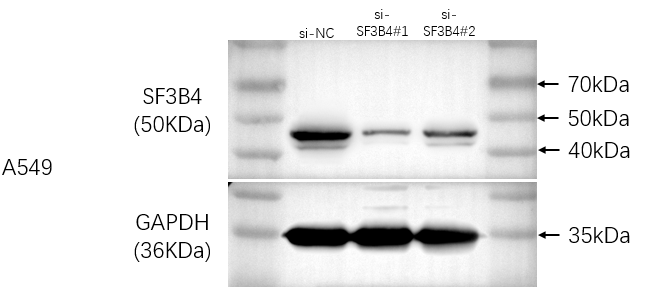


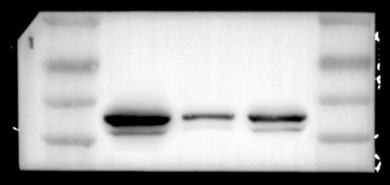


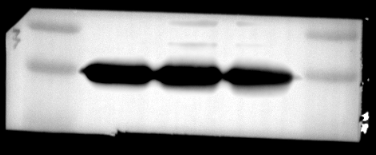


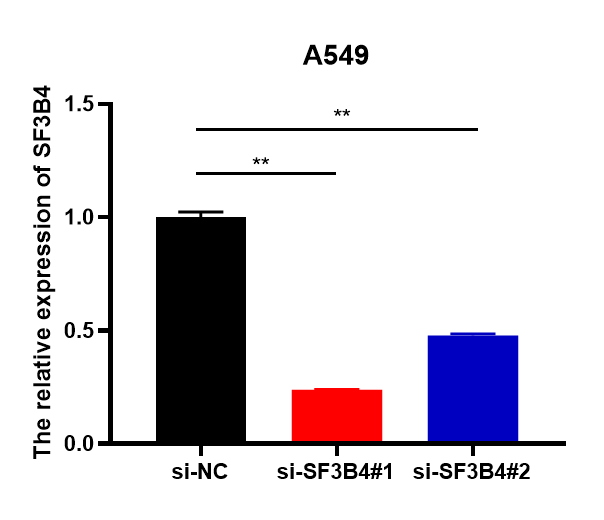


**Figure 2A**


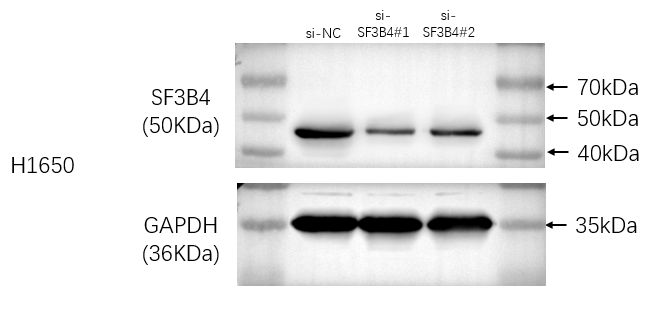


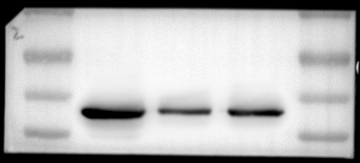


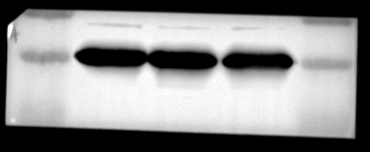


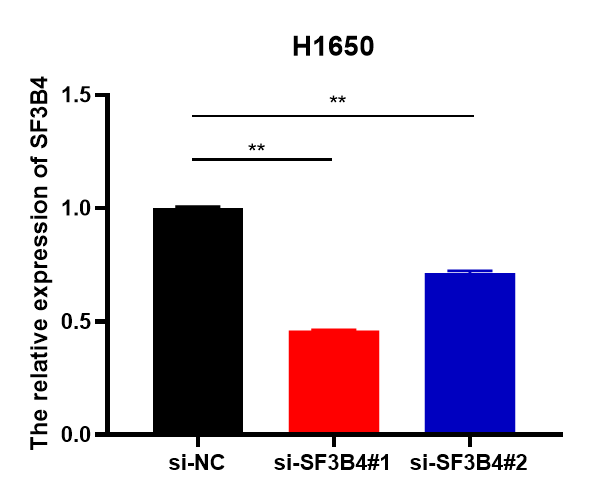


**Figure 4H**


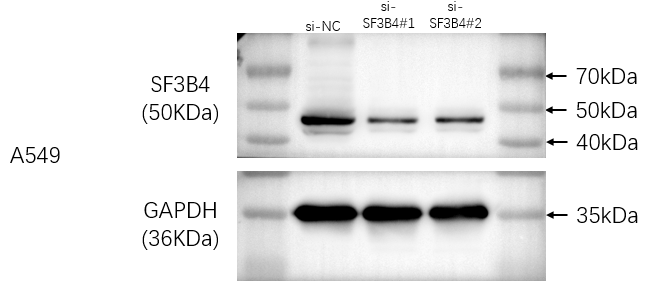


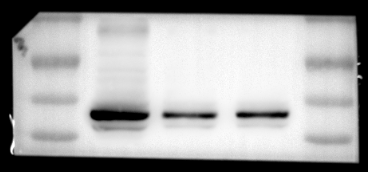


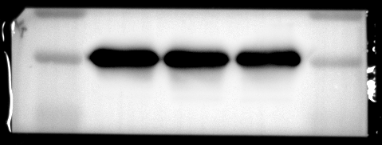


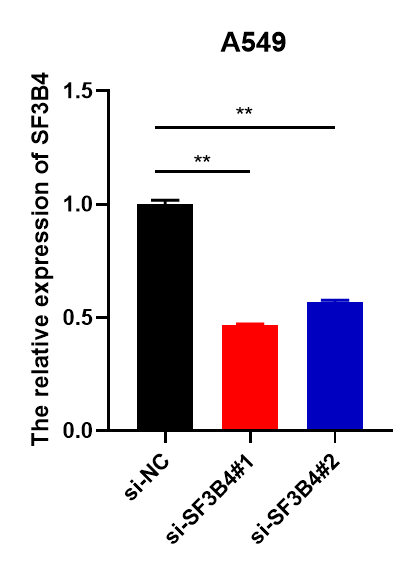


**Figure 4H**


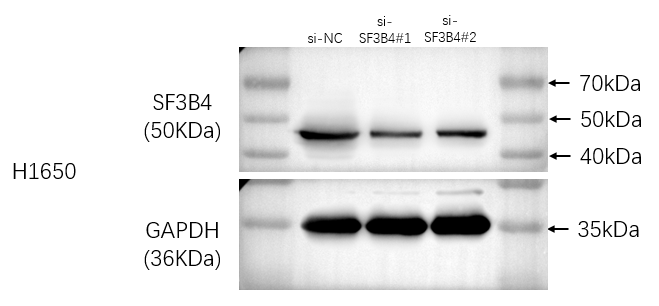


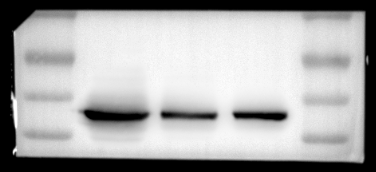


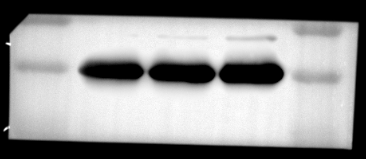


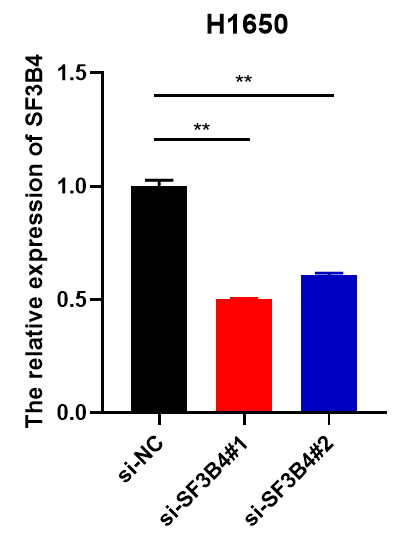


**Figure 4H**


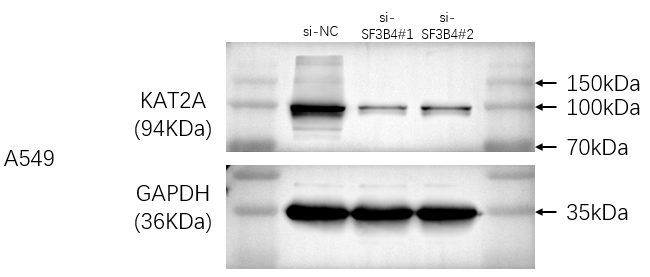


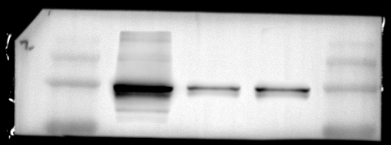


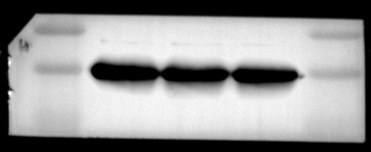


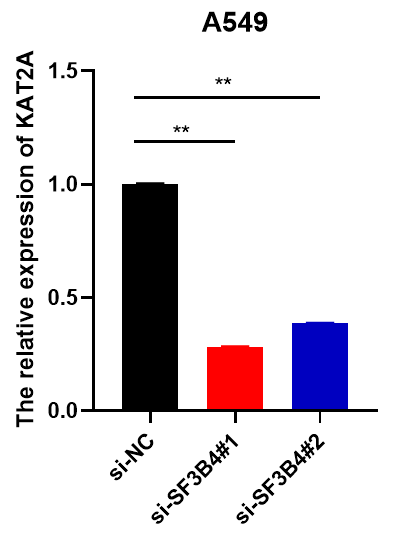


**Figure 4H**


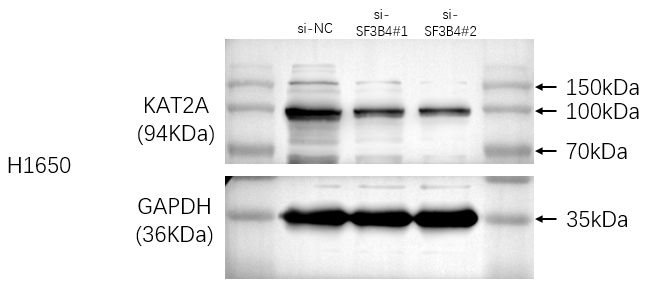


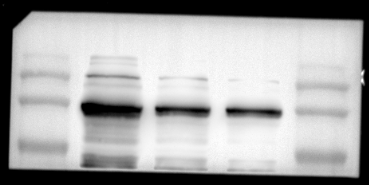


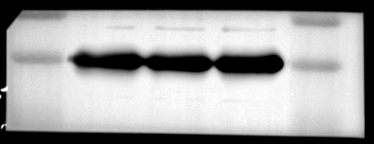


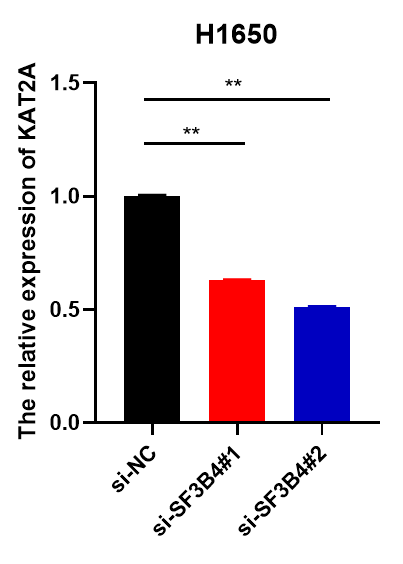


**Figure 6B**


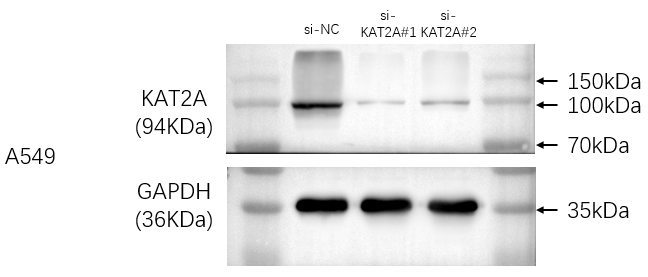


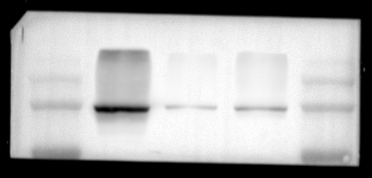


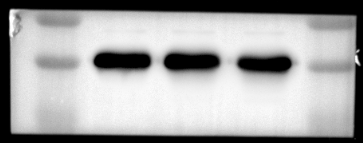


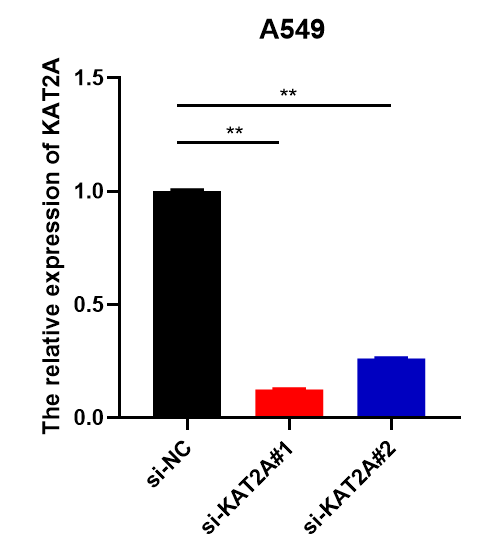


**Figure 6B**


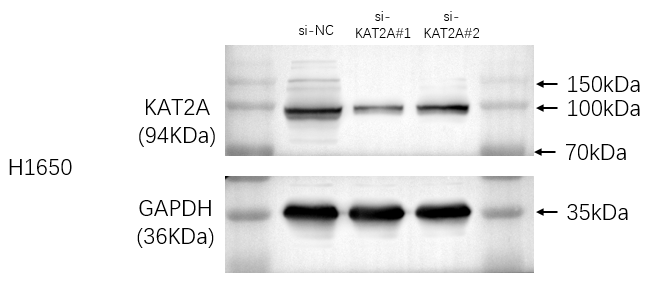


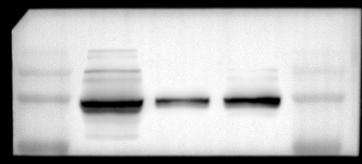


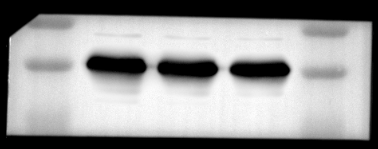


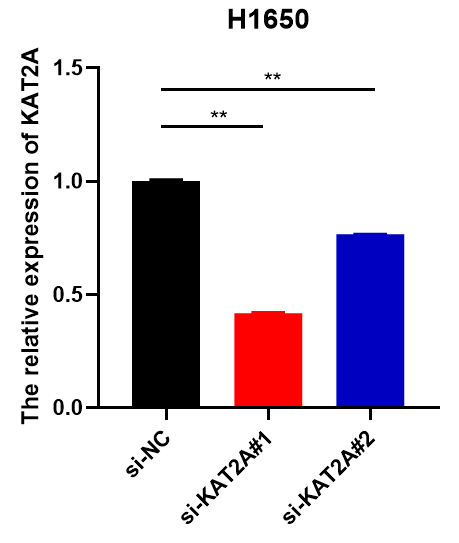


**Figure 7B**


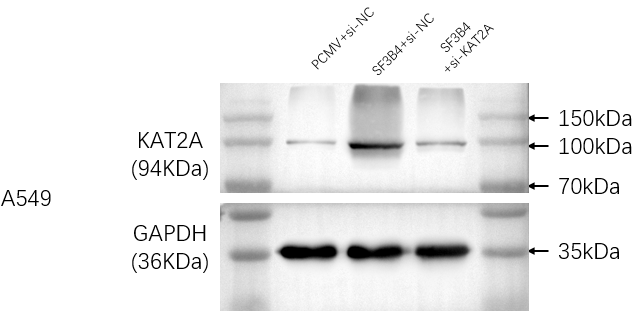


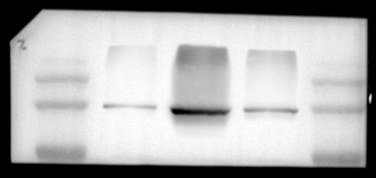


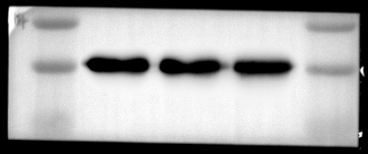


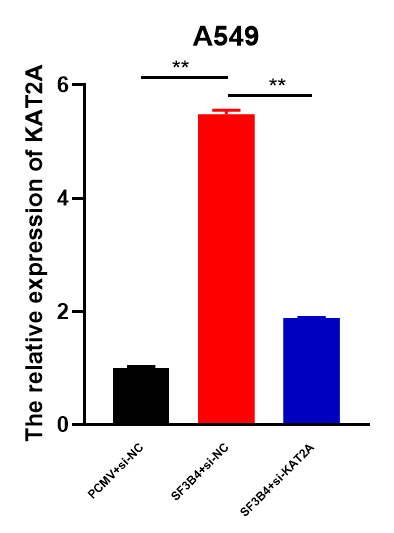


**Figure 7B**


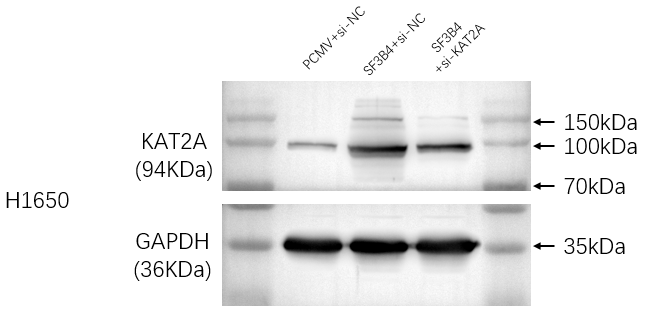


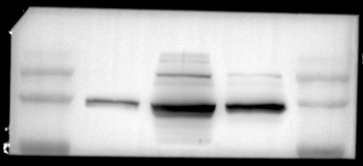


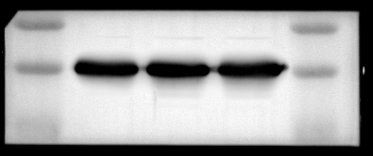


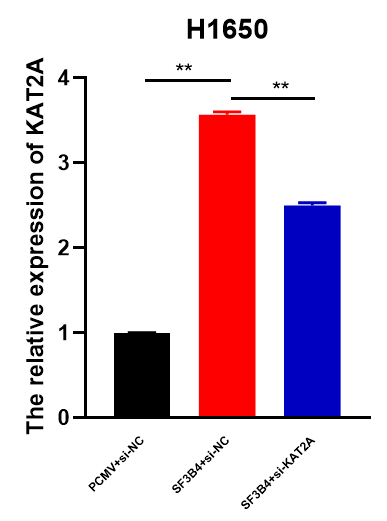


**Figure 5C**

**A549 unspliced**

Original gel electrophoresis photograph
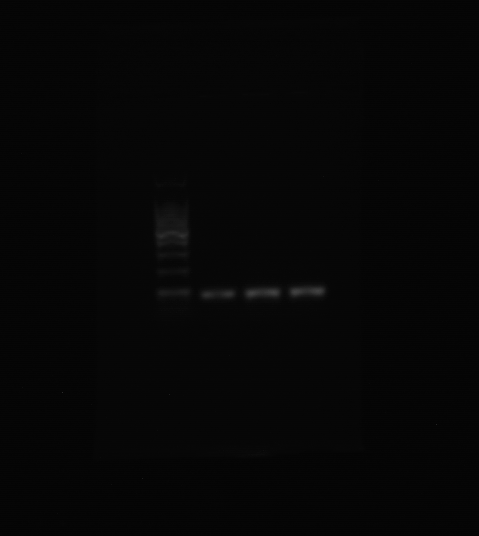


In order to facilitate observation, the black and white image of the photo is reversed


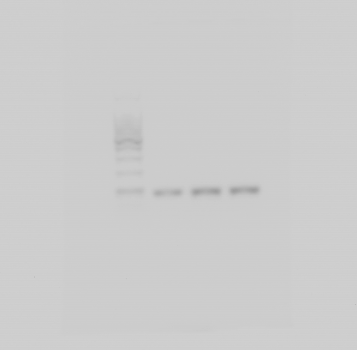


**Figure 5C**

**A549 spliced**

Original gel electrophoresis photograph


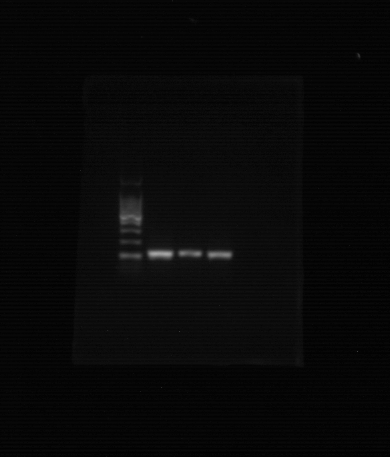


In order to facilitate observation, the black and white image of the photo is reversed


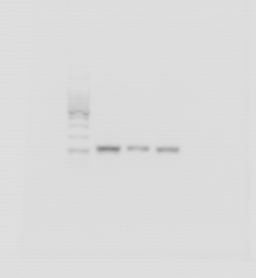


**Figure 5C**

**A549 GAPDH**

Original gel electrophoresis photograph


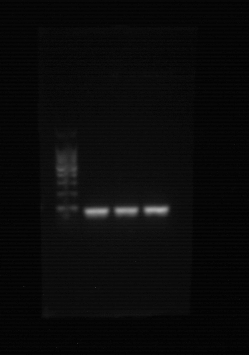


In order to facilitate observation, the black and white image of the photo is reversed


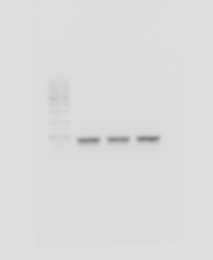


**Figure 5C**

**H1650 unspliced**

Original gel electrophoresis photograph


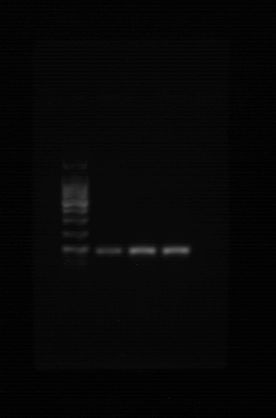


In order to facilitate observation, the black and white image of the photo is reversed


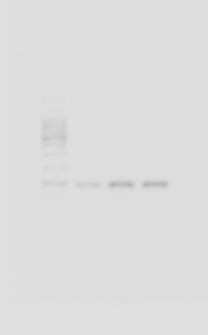


**Figure 5C**

**H1650 spliced**

Original gel electrophoresis photograph


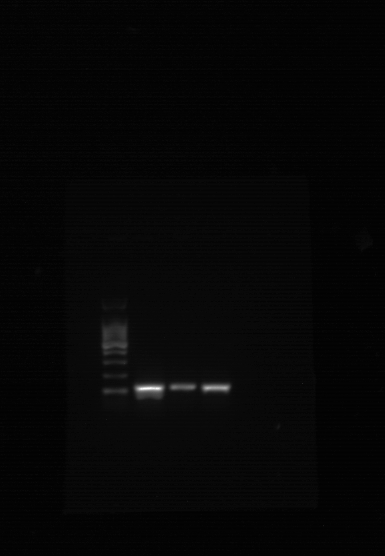


In order to facilitate observation, the black and white image of the photo is reversed


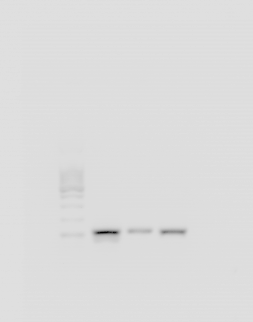


**Figure 5C**

**H1650 GAPDH**

Original gel electrophoresis photograph


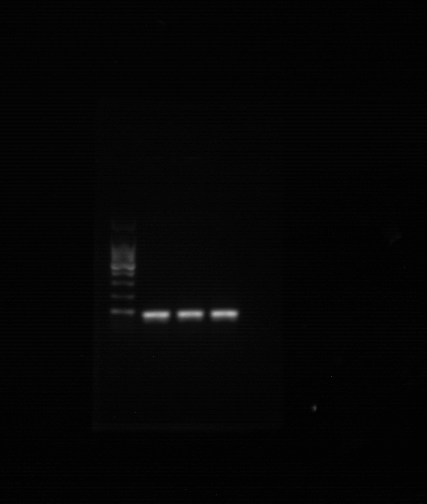


In order to facilitate observation, the black and white image of the photo is reversed


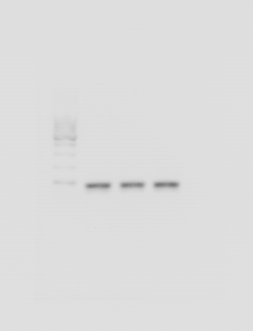

Supplement: Supplementary file 1 — Supplementary Information. [file 41598_2023_50606_MOESM1_ESM.doc]
